# Notch signaling regulates *Hey2* expression in a spatiotemporal dependent manner during cardiac morphogenesis and trabecular specification

**DOI:** 10.1038/s41598-018-20917-w

**Published:** 2018-02-08

**Authors:** Lianjie Miao, Jingjing Li, Jun Li, Xueying Tian, Yangyang Lu, Saiyang Hu, David Shieh, Ryan Kanai, Bo-yang Zhou, Bin Zhou, Jiandong Liu, Anthony B. Firulli, James F. Martin, Harold Singer, Bin Zhou, Hongbo Xin, Mingfu Wu

**Affiliations:** 1 Department of Molecular and Cellular Physiology, Albany Medical College, Albany, 12208 NY USA; 2 Institute of Translational Medicine, Nanchang University, Nanchang, China; 3 School of Life Sciences, Nanchang University, Nanchang, China; 4 The State Key Laboratory of Cell Biology, Shanghai Institute of Biochemistry and Cell Biology, Chinese Academy of Sciences, University of Chinese Academy of Sciences, Shanghai, 200031 China; 5 Department of Genetics, Albert Einstein College of Medicine of Yeshiva University, Bronx, New York, 10461 USA; 6 Department of Pathology and Laboratory Medicine, McAllister Heart Institute, University of North Carolina at Chapel Hill, Chapel Hill, NC 27599 USA; 7 Riley Heart Research Center, Wells Center for Pediatric Research, Departments of Pediatrics and Medical and Molecular Genetics, Indiana University, Indianapolis, IN 46202 USA; 8 Department of Molecular Physiology and Biophysics, Baylor College of Medicine, Houston, TX USA

## Abstract

*Hey2* gene mutations in both humans and mice have been associated with multiple cardiac defects. However, the currently reported localization of *Hey2* in the ventricular compact zone cannot explain the wide variety of cardiac defects. Furthermore, it was reported that, in contrast to other organs, Notch doesn’t regulate *Hey2* in the heart. To determine the expression pattern and the regulation of *Hey2*, we used novel methods including RNAscope and a *Hey2*^*CreERT2*^ knockin line to precisely determine the spatiotemporal expression pattern and level of *Hey2* during cardiac development. We found that *Hey2* is expressed in the endocardial cells of the atrioventricular canal and the outflow tract, as well as at the base of trabeculae, in addition to the reported expression in the ventricular compact myocardium. By disrupting several signaling pathways that regulate trabeculation and/or compaction, we found that, in contrast to previous reports, Notch signaling and *Nrg1/ErbB2* regulate *Hey2* expression level in myocardium and/or endocardium, but not its expression pattern: weak expression in trabecular myocardium and strong expression in compact myocardium. Instead, we found that FGF signaling regulates the expression pattern of *Hey2* in the early myocardium, and regulates the expression level of *Hey2* in a *Notch1* dependent manner.

## Introduction

Hey2, together with Hey1, HeyL and their presumptive *Drosophila* homologue, dHey, is a member of a subfamily of hairy-related basic helix-loop-helix (bHLH) transcription factors^1,2^, that are implicated in cell fate determination and boundary formation^3^. *Hey2* mutations in both humans and mice cause a variety of cardiac morphogenetic defects, as well as a cardiomyocyte maturation defect. In humans, non-synonymous sequence changes in *Hey2* correlate with atrioventricular septal defects and other cardiac defects^4,5^, and *Hey2* duplication contributes to both congenital heart defects and neurodevelopmental defects^6^. Similarly, *Hey2* knockout mice display defects including atrioventricular valvular defects, pulmonary stenosis, Tetralogy of Fallot, tricuspid atresia, and abnormal cardiac hemodynamics^7–9^, indicating that *Hey2* is an essential regulator of cardiac morphogenesis and cardiac function. Furthermore, genome-wide association study associated *Hey2* mutation with Brugada syndrome, a rare cardiac arrhythmia disorder in humans^10^.

Despite profound cardiac defects caused by *Hey2* mutation in both humans and mice, the current knowledge of the *Hey2* expression pattern—that *Hey2* is expressed in the compact zone of the myocardium—cannot fully explain this broad range of congenital defects. It is unclear how *Hey2* enrichment in the ventricular compact myocardium could contribute to atrioventricular morphogenesis and semilunar valvular morphogenesis. Using an RNAscope, an assay that enables single mRNA molecule detection and is compatible with immunofluorescent staining, we were able to determine the cell type- and developmental stage-specific expression pattern of *Hey2* at the level of single mRNA molecule. Specifically, we found that, in addition to the previously reported expression in the ventricular compact myocardium and interventricular septum, *Hey2* is also expressed in the endocardial cells of the atrioventricular canal (AVC), the outflow tract (OFT), and at the base of trabeculae, as well as in pro-epicardial cells and epicardial cells. The expressional level and pattern of *Hey2* examined by the RNAScope were also consistent with the expression pattern determined by the *Hey2*^*CreERT2*^ knock-in mouse line.

*Hey2* functions in cell fate specification and cardiomyocyte maturation. *Gridlock/Hey2* is involved in adjudicating an arterial versus venous cell fate decision during the assembly of the first embryonic artery in zebrafish^11^. In mice, cardiac specific *Hey2* knockout hearts display ectopic atrial gene expression^12,13^. *Hey2* null cardiomyocytes displayed abnormal mitochondria, abnormal accumulation of glycogen particles, and disorganized myofibrils based on transmission electron microscope analysis^8^. Expression levels of β-MHC and ANF genes in the *Hey2* knockout are increased^8^. The phenotypes of *Hey2* knockout heart indicate that Hey2 might play roles in cardiomyocyte differentiation and maturation.

Previous work has suggested that trabecular cardiomyocytes, which take the major responsibility for pumping during the early stages of cardiac development, are more differentiated than the cardiomyocytes of the compact zone^14^. The facts that *Hey2* is expressed in the cardiomyocytes of the compact myocardium and that *Hey2* disruption results in a larger and wider sarcomere suggest that *Hey2* might repress cardiomyocyte maturation and might serve as a marker for less differentiated and/or less mature cardiomyocytes. Previously, single cell lineage tracing studies revealed that asymmetric distribution of *Hey2* during cardiomyocyte oriented cell division might contribute to the differential expression of *Hey2* in compact and trabecular cardiomyocytes^15^. However, the signaling pathways that regulate the *Hey2* expression pattern and its asymmetric distribution in a perpendicular oriented cell division during trabecular initiation are unknown. To identify the signaling pathways that regulate the differential expression levels of *Hey2* in the compact zone and the trabecular zone, we assessed the expression of *Hey2* in various mutants that are defective in several pathways implicated in trabeculation and compaction, including *Nrg1/ErbB2/4* and *Notch1*. We found that these mutants showed decreased expression levels of *Hey2*, but displayed normal enrichment and repression of *Hey2* in the compact zone and trabecular zone, respectively. FGF ligand stimulation changes the expression pattern of *Hey2* in the ventricles temporally in both *in vivo* and *ex vivo* models, resulting in more trabeculation. We further found that FGF2 regulates the expression level of *Hey2* in a *Notch1* dependent manner.

## Results

### *Hey2* is expressed in the endocardial cells of the AVC, OFT and the base of trabeculae in addition to its known localization to the compact zone

**Figure 1:**
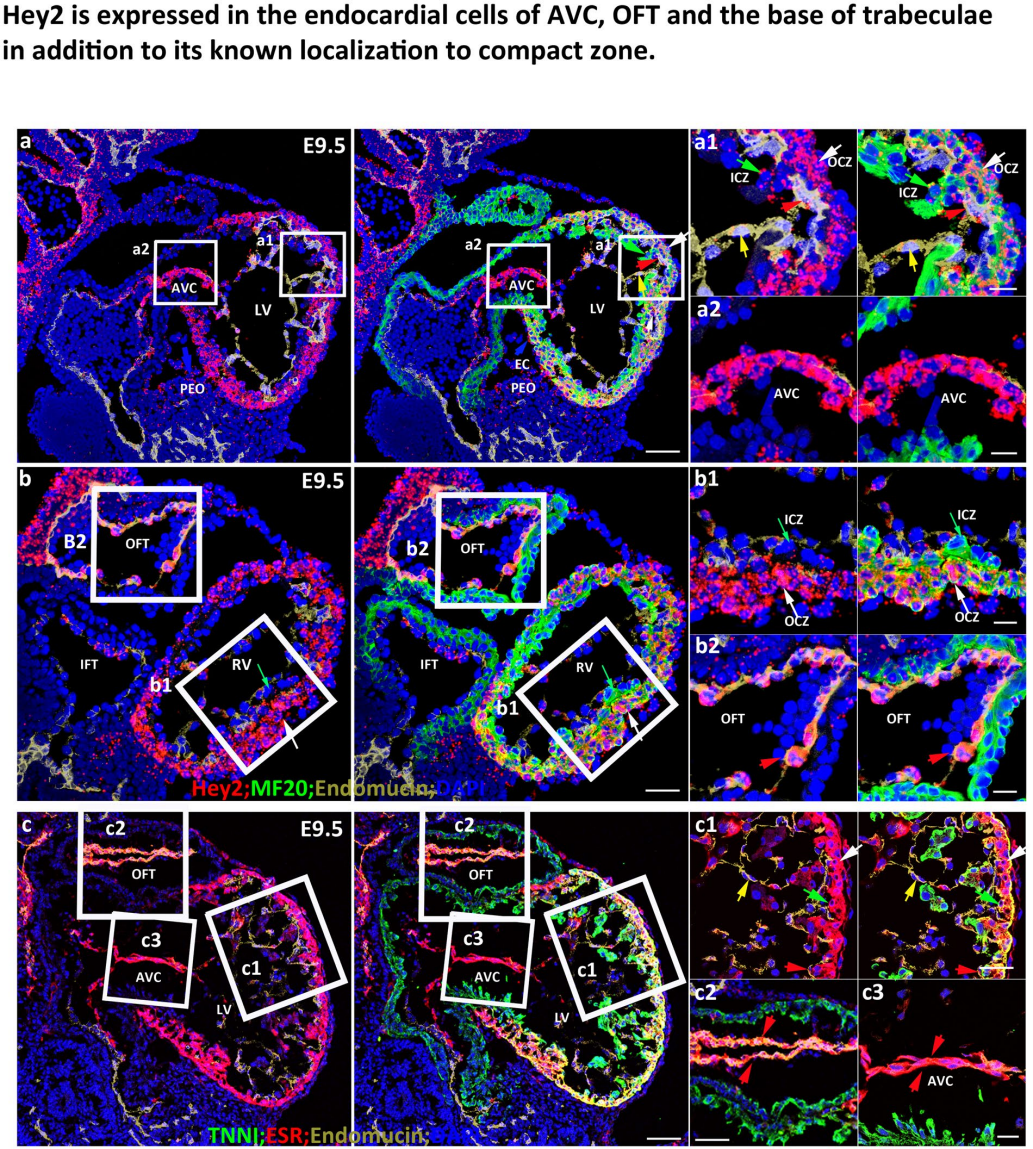
Hey2 is expressed in the endocardial cells of the AVC, OFT and the base of trabeculae, in addition to its known localization to the compact zone. (a and b) *Hey2* expression pattern in E9.5 control heart. Box a1,a2 and Box b1,b2 in Figures a,b were zoomed to a1,a2 and b1,b2 on the right. White and green arrows in a1,b1 point to the compact zone (OCZ) and inner compact zone (ICZ), respectively. Red and yellow arrows in a1 point to endocardial cells that attach or do not attach to cardiomyocytes, respectively. The red arrow in b2 points to endocardial cells of the OFT. Blue arrow in a points to pro-epicardial organ (PEO). **(c)** The expression pattern of *Hey2* via ESR staining using the *Hey2*^*CreERT2*^ mouse line. White and green arrows in c1 point to the outer and inner compact zone, respectively. Red and yellow arrows in c1 point to endocardial cells that attach or do not attach to cardiomyocytes, respectively. The red arrow in c1 points to endocardial cells of the ventricle. LV: Left ventricle; RV: Right ventricle; AVC: Atrioventricular canal; EC: Epicardial cell; PEO: Pro-epicardial organ; OFT: Outflow tract; IFT: Inflow tract. Scale bars in (**a–c**) are 50 μm and 10 μm in zoomed boxes. Representative pictures from at least three embryos of each genotype at different ages were shown.

Previous *in situ* hybridization (ISH) studies using whole embryos or tissue sections demonstrated that *Hey2* is expressed in the compact zone, AV cushion, and ventricular septum^1,16^. However, harsh treatment in traditional ISH protocols prevents successful simultaneous immunofluorescence staining, which is necessary to accurately identify the cell types that express *Hey2*. In this study, we used the RNAscope, which can detect single mRNA molecules and allows for ISH and immunofluorescence staining in the same samples^15^, and a *Hey2*^*CreERT2*^ knockin mouse line^17^, to determine the expression level and pattern of *Hey2* and found a broader expression pattern of *Hey2* than previously reported. In both left and right ventricles, *Hey2* is enriched in the outer compact zone (OCZ), and is weakly expressed in the inner compact (ICZ) and trabecular zones (Fig. 1a,a1,b and b1). In addition to its expression in the outer compact zone, *Hey2* is robustly expressed in endocardial cells of the atrioventricular canal (AVC) (Fig. 1a and a2) and outflow tract (OFT) (Fig. 1b and b2) at E9.5. Furthermore, *Hey2* is weakly expressed in the endocardial cells at the base of trabeculae, and the endocardial cells that closely abut the cardiomyocytes contains 6.25 dots/section/cell (n = 6), which is relatively greater than the *Hey2* expression in endocardial cells that are not adjacent to the cardiomyocytes, which contain 3.20 dots (n = 6) (Fig. 1a and a1). We also examined the *Hey2* expression pattern by staining with estrogen receptor (ESR) using E9.5 hearts from *Hey2*^*CreERT2*^ knockin embryos, and consistently *Hey2* was strongly expressed in the compact zone and in the endocardial cells of the AVC and OFT (Fig. 1c,c2 and c3), and weakly expressed in the cardiomyocytes of trabecular zone and the ventricular endocardial cells (Fig. 1c). However, via the ESR staining, we did not observe the differential expression of *Hey2* in outer and inner compact zone, and its differential expression in ventricular endocardial cells at different regions cannot be distinguished (Fig. 1a–c), which might be due to the detection threshold of ESR proteins distingguishable by antibody-mediated immunostaining.

### *Hey2* is expressed in the mesenchymal cells of the AVC and OFT

**Figure 2:**
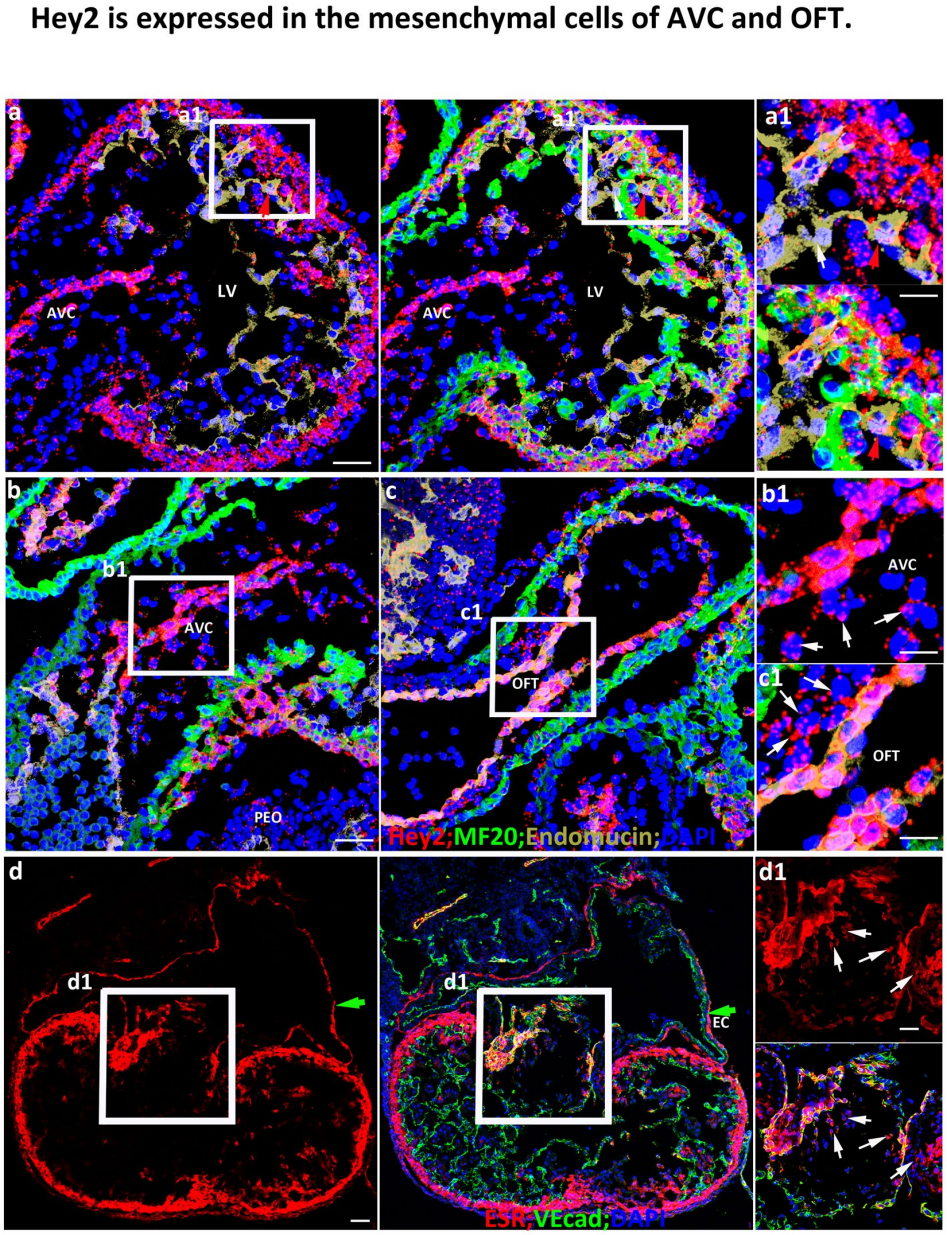
Hey2 is expressed in the mesenchymal cells of AVC and OFT. **(a–c)** *Hey2* expression pattern in an E10.5 control heart via RNAScope. The box regions in (**a**–**c**) are zoomed and put to the right. The red arrow in a1 points to a cell that closely attaches to cardiomyocytes and displayed more *Hey2* molecules than a cell in a1 that does not attach to cardiomyocytes, as indicated by a white arrow. White arrows in b1 and c1 point to mesenchymal cells that are derived from the AVC and OFT, respectively. **(d)** The expression pattern of *Hey2* via ESR staining using the *Hey2*^*CreERT2*^ mouse line at E11.5. Green arrows in d point to epicardial cells and white arrows in d1 point to the mesenchymal cells that are derived from the endocardial cells of AVC. LV: Left ventricle; RV: Right ventricle; AVC: Atrioventricular canal; EC: Epicardial cell; PEO: Pro-epicardial organ; OFT: Outflow tract. Scale bars in (**a**–**d**) are 50 μm and 10 μm in zoomed boxes. Representative pictures from at least three embryos of each genotype at different ages were shown.

Consistently with its expression pattern at E9.5, *Hey2* is highly expressed in the outer compact zone, endocardial cells of the AVC and OFT, and weakly expressed in ventricular endocardial cells in E10.5 hearts (Fig. 2a–c). The endocardial cells that closely abut against the cardiomyocytes display more *Hey2* compared to the cells that are not adjacent to cardiomyocytes (Fig. 2a and a1). *Hey2* is also expressed in the cells between endocardial cells and cardiomyocytes, which are mesenchymal cells derived from endocardial cells of the AVC (Fig. 2b and b1) and OFT (Fig. 2c and c1), at a lower intensity compared to the endocardial cells of the AVC and OFT. Via the *Hey2Cre*^*ERT2*^, we found that *Hey2* is also expressed in the endocardial cells and mesenchymal cells of AVC and OFT cushion (Fig. 2d and d1). The expression of *Hey2* in these endocardial cells and cushion tissues might suggest that *Hey2* is involved in endocardial cell epithelial-mesenchymal transition (EMT) and that *Hey2* disruption leads to the valvular morphogenesis defects previously reported in *Hey2* knockouts^16^. In addition to its expression in the aforementioned endocardial cells, we also found that *Hey2* is weakly expressed in pro-epicardial cells and epicardial cells (Figs 1a and 2d).

### Notch signaling regulates Hey2 expression in ventricular endocardial cells

**Figure 3:**
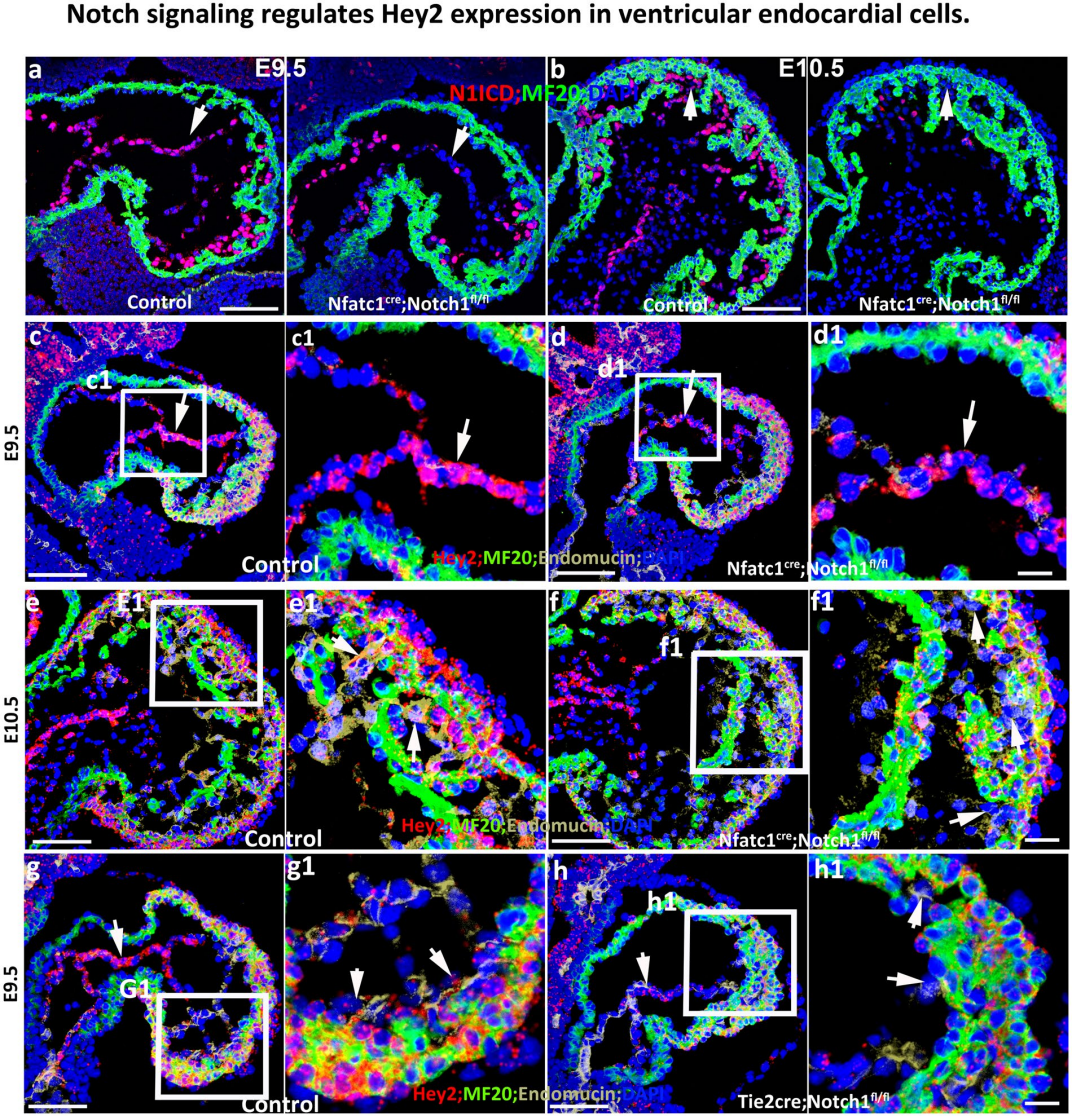
Notch signaling regulates Hey2 expression in ventricular endocardial cells. **(a and b)** N1ICD staining in control and *Nfatc1*^*cre*^*; Notch1*^*fl/fl*^ hearts at E9.5 and E10.5. White arrows show reduced NICD expression in a knockout compared to control in a&b. (c and d) *Hey2* expression in control and *Nfatc1*^*cre*^*; Notch1*^*fl/fl*^ hearts at E9.5. Endocardial cells pointed by white arrows in d and d1 show reduced Hey2 expression compared to c&c1. (e and f) *Hey2* expression in control and *Nfatc1*^*cre*^*; Notch1*^*fl/fl*^ hearts at E10.5. Endocardial cells pointed by white arrows in f1 show reduced Hey2 expression compared to e1. (g and h) Hey2 expression in control and Tie2cre; Notch1^fl/fl^ hearts at E9.5. White arrows in f1 and h1 indicate reduced expression of *Hey2* in ventricular endocardial cells and AVC endocardial cells compared to their controls (e1 and g1). The box regions in (**c**–**h**) are zoomed to separate pictures that are labeled with the same name of the box. Scale bars in (**a**–**h**) are 100 μm and 10 μm in zoomed boxes. Representative pictures from at least three embryos of each genotype at different ages were shown.

Previous work has shown that *Hey2* expression is altered in *Dll1* or *Notch1* knockout mice during somitogenesis^18,19^. However, during cardiac morphogenesis, studies have demonstrated that Notch Intracellular Domain (NICD) overexpression activated *Hey1* but not *Hey2* expression in the heart using the NICD transgenic line^20^. Furthermore, *Notch2* knockout mice displayed normal expression patterns of *Hey1* and *Hey2* in the myocardium^21^ and *Rbpjk* global knockout embryos did not alter the expressional level of *Hey2* in the heart based on Q-PCR^22^. These reports indicate that Notch signaling does not regulate *Hey2* expression in the heart. However, it is possible that Notch signaling regulates *Hey2* expression in some cell types such as endocardial cells that cannot be detected by whole heart RNA quantification and traditional ISH. To test this possibility, we examined the expression of *Hey2* in different mutants via RNAscope coupled with immunofluorescence staining. We used *Nfatc1*^*Cre/*+^, which is specifically expressed in the endocardial cells^23^, to mediate *Notch1* deletion, and found reduced NICD expression (Fig. 3a and b). *Nfatc1*^*Cre/*+^*; Notch1*^*fl/fl*^ knockout hearts showed a slight reduction in *Hey2* in the endocardial cells of the AVC at E9.5, but did not show obvious reduction of *Hey2* in the compact zone (Fig. 3c,c1,d and d1). Due to the incomplete deletion of *Notch1* at early stages such as E9.5 (Fig. 3a), we examined the knockouts at E10.5 and consistently found that *Hey2* expression was reduced in the endocardial cells of AVC and also in the endocardial cells that closely interact with cardiomyocytes at the base of trabeculae, while *Hey2* expression in the compact zone was not obviously altered (Fig. 3e,e1,f and f1). *Hey2* expression in the endocardial cells of the OFT was not changed in the *Nfatc1*^*Cre/*+^*; Notch1*^*fl/fl*^ (Data not shown). The difference in expression of *Hey2* between control and knockout hearts was not as large as expected, which might be due to late expression of Cre in the *Nfatc1*^*Cre/*+^ mouse (Fig. 3a,b). Therefore, we used *Tie2Cre*, which is expressed starting at E7.5, to delete *Notch1* in endocardial cells. This knockout died at around E10.5 and displayed a trabeculation defect as previously reported^24^. *Hey2* expression in the endocardial cells of this knockout was significantly reduced at E9.5 (Fig. 3g,g1,h and h1). To determine if other Notch receptors in addition to the *Notch1* receptor regulate *Hey2* in endocardial cells, we examined the expression of *Hey2* in *Tie2Cre*-mediated *Rbpjk* knockout hearts. Surprisingly, the *Hey2* expression level in the endocardial cells of the *Tie2Cre; Rbpjk*^*fl/fl*^ knockout was not obviously different from that of the *Tie2Cre; Notch1*^*fl/fl*^ knockout (Suppl. Figure 1a,b), suggesting that Notch1 is the major receptor in endocardial cells that regulates *Hey2* expression. The incomplete abolishment of *Hey2* expression in the endocardial cells, especially those of the AVC in *Tie2Cre; Rbpjk*^*fl/fl*^ knockouts, indicates that an *Rbpjk*-independent signaling pathway also regulates *Hey2* expression in the endocardium. We examined total *Hey2* in control and *Tie2Cre; Rbpjk*^*fl/fl*^ knockout hearts via Q-PCR and found that the knockouts displayed higher levels of *Hey2* (Suppl. Figure 1c), possibly due to a relatively smaller trabecular zone and a larger compact zone, where Hey2 is enriched.

### Notch signaling regulates the expression level but not the pattern of *Hey2* in the myocardium

**Figure 4:**
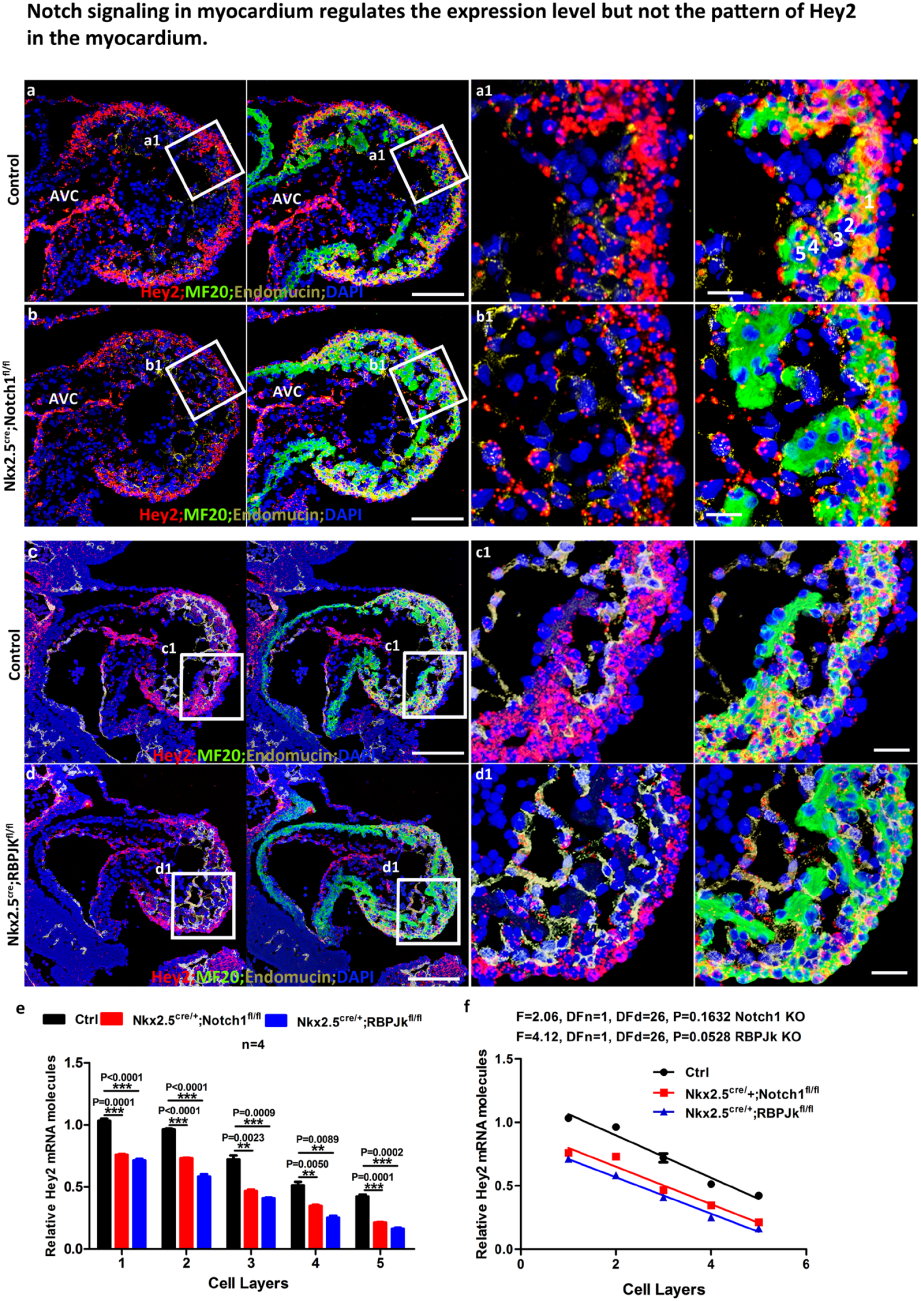
Notch signaling in myocardium regulates the expression level but not the pattern of *Hey2* in the myocardium. (a and b) *Hey2* expression in E9.5 control and *Nkx2*.*5*^*cre*^*; Notch1*^*fl/fl*^ hearts. The white numbers in a1 indicate the cell layers from the 1^st^ to the 5^th^ layer of ventricular myocardium and the number of *Hey2* expression in different layers were quantified and presented in (**e)**. (c and d) *Hey2* expression in E9.5 control and *Nkx2*.*5*^*cre*^*; Rbpjk*^*fl/fl*^ hearts. The box regions in (**a**–**d**) are zoomed to separate pictures that are labeled with the same name of the box. **(e**) *Hey2* enrichment index. The average number of *Hey2* molecules from 10 cells from each layer was quantified, and their ratio to layer #1, which is considered as 1, was presented in (**e**) Quantification of *Hey2* expression at different layers in *Nkx2*.*5*^*cre*^*; Notch1*^*fl/fl*^, and *Nkx2*.*5* ^*cre*^*; RBPJk*^*fl/fl*^ hearts were shown. Expression level of *Hey2* in different layer of cells of different genotypes were quantified and compared via student’s t-test. (**f**) The expression patterns of *Hey2* in different treatments were analyzed by linear regression comparison and they were not significantly different from the control based on the lineage regression comparisons. Scale bars in (**a**–**h**) are 100 μm and 10 μm in zoomed box. Representative pictures from at least three embryos of each genotype or treatment at different ages were shown.

Echocardiographic studies of *Hey2* knockout mice revealed reduced left ventricular contractile function^8^. It is speculated that the presence of abnormal cardiomyocytes accounts for the decreased left ventricle fractional shortening, indicating the importance of *Hey2* in compact zone cardiomyocyte maturation. The signaling pathways that regulate the enrichment of *Hey2* in the compact zone and the low abundance in the trabecular zone have not been reported before. Our data showed that *Notch1* or *Rbpjk* deletion in endocardial cells via *Nfatc1*^*Cre/*+^ or *Tie2Cre* did not affect the *Hey2* enrichment in the compact zone (Fig. 3), indicating that Notch signaling in the endocardium does not regulate *Hey2* in the myocardium non-autonomously—as it does with many other factors in the myocardium^24,25^. We then investigated if *Notch1* and *Rbpjk* regulate *Hey2* in the compact zone autonomously by examining *Hey2* expression in the myocardium of both *Nkx2*.*5*^*Cre/+*^; *Notch1*^*fl/fl*^ and *Nkx2*.*5*^*Cre/+*^; *Rbpjk*^*fl/fl*^ knockouts. *Nkx2*.*5*^*Cre/+*^ mediated *Notch1* or *Rbpjk* deletion did not change the *Hey2* expression pattern between the control and knockout at both E9.5 and E12.5 (Fig. 4a–d, Suppl. Figure 2a,b). We then quantified the number of *Hey2* molecules within cells in different layers of the myocardium with the enrichment index of *Hey2* in different layers determined by their relative numbers of *Hey2* molecules (Fig. 4a,e). We found that the intensity of *Hey2* expression in the compact zone of the knockout is reduced compared to the control in both knockouts (Fig. 4a–e), while its intensity in other regions, such as endocardial cells, was not obviously affected (Fig. 4a–d). The *Nkx2*.*5*^*Cre/+*^; *Rbpjk*^*fl/fl*^ heart showed slightly reduced *Hey2* expression based on Q-PCR (Suppl. Figure 2c). We also examined whether the *Notch1* deletion would disrupt the expression pattern of *Hey2* in the compact and trabecular zone. The *Hey2* expression pattern in the knockouts was compared to that of the controls by linear regression comparison, and we found that the expression pattern among the control and knockouts are not significantly different based on the slopes (Fig. 4e,f). Consistently, when *ex vivo* cultured hearts were treated with DAPT, a γ-secretase inhibitor which prevents Notch activation, the expression pattern of *Hey2* was not changed, although the level was slightly reduced (Data not shown). These data suggest that Notch signaling mildly regulates the expression level of *Hey2*, but not its expression pattern in the myocardium.

### The Nrg1/ErbB2 pathway does not regulate *Hey2* expression pattern

**Figure 5:**
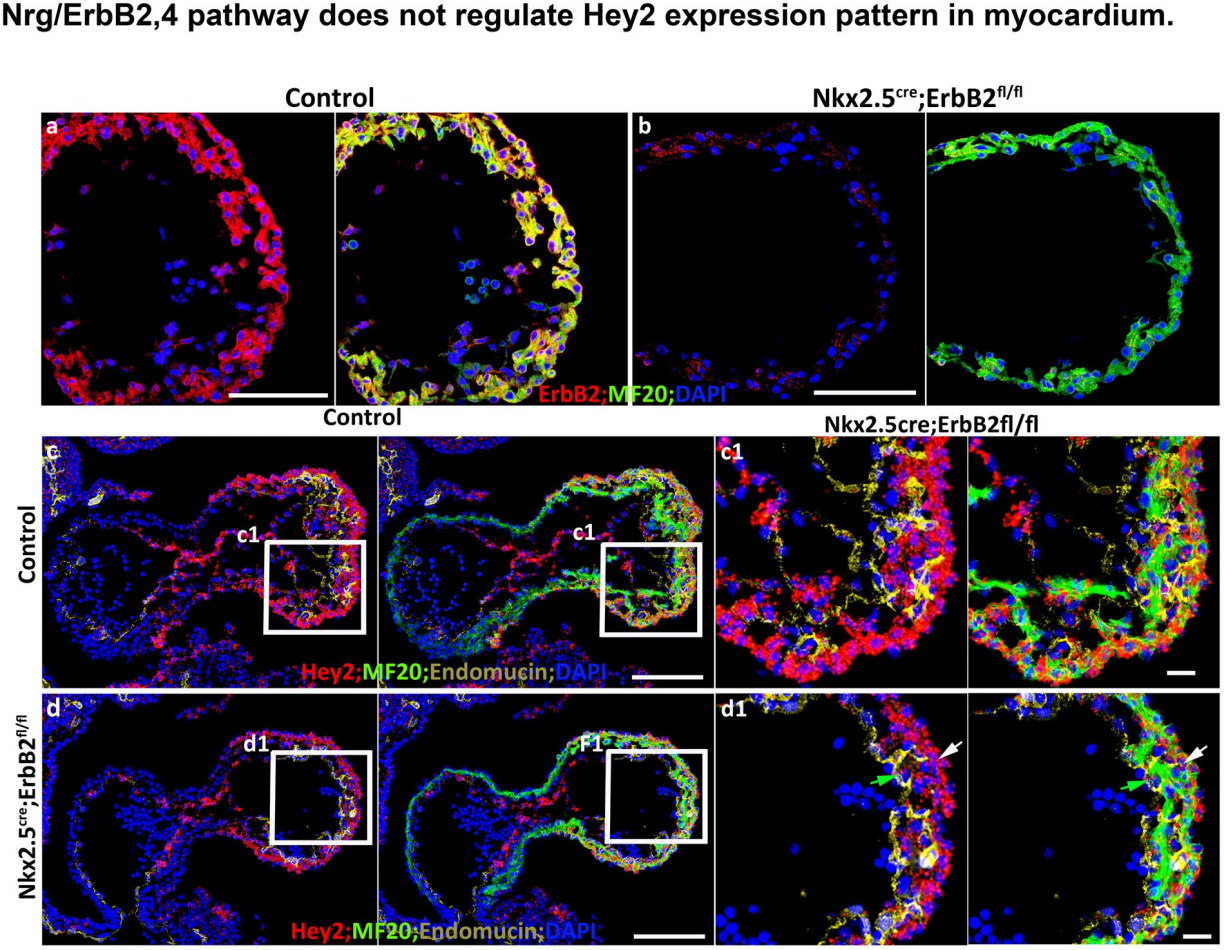
*Nrg/ErbB2*,*4* pathway does not regulate *Hey2* expression pattern. (a and b) *ErbB2* deletion in *Nkx2*.*5*^*cre*^*; ErbB2*^*fl/fl*^ at E9.5 indicated by the absence of ErbB2 immunostaining in the knockout. (c and d) *Hey2* expression in E9.5 control and *Nkx2*.*5*^*cre*^*; ErbB2*^*fl/fl*^ hearts. The box regions in **c,d** are zoomed to separate pictures that are labeled with the same name of the box. Scale bars in (**a**–**d**) are 100 μm. Representative pictures from at least three embryos of each genotype were shown.

We were then interested in identifying the signaling pathways that regulate the expression pattern of *Hey2* in the compact zone. Previous work has shown that global deletion of the receptors or ligand of the Nrg1/ErbB2,4 pathway abrogates trabecular formation^26–28^. We used the *Nkx2*.*5*^*Cre/+*^ line to delete *ErbB2* (Fig. 5a,b). The *Nkx2*.*5*^*Cre/+*^*; ErbB2*^*fl/fl*^ knockout hearts displayed a lower proliferation rate and thinner compact zone (Data not shown). Previous work has shown that Notch1 signaling regulates cardiomyocyte differentiation through Nrg1/ErbB2,4^24^. We hypothesized that Nrg1/ErbB2,4 might regulate *Hey2* expression in the compact zone. We examined the expression of *Hey2* in the *Nkx2*.*5*^*Cre/+*^*; ErbB2*^*fl/fl*^ heart and found that the *Hey2* expression level was slightly reduced (Suppl. Figure 3a), but its expression pattern and relative enrichment in the compact zone was not affected (Fig. 5c,d), suggesting that Nrg1/ErbB2,4 does not regulate the *Hey2* expression pattern.

### FGF signaling regulates *Hey2* expression pattern in the ventricles temporally

**Figure 6:**
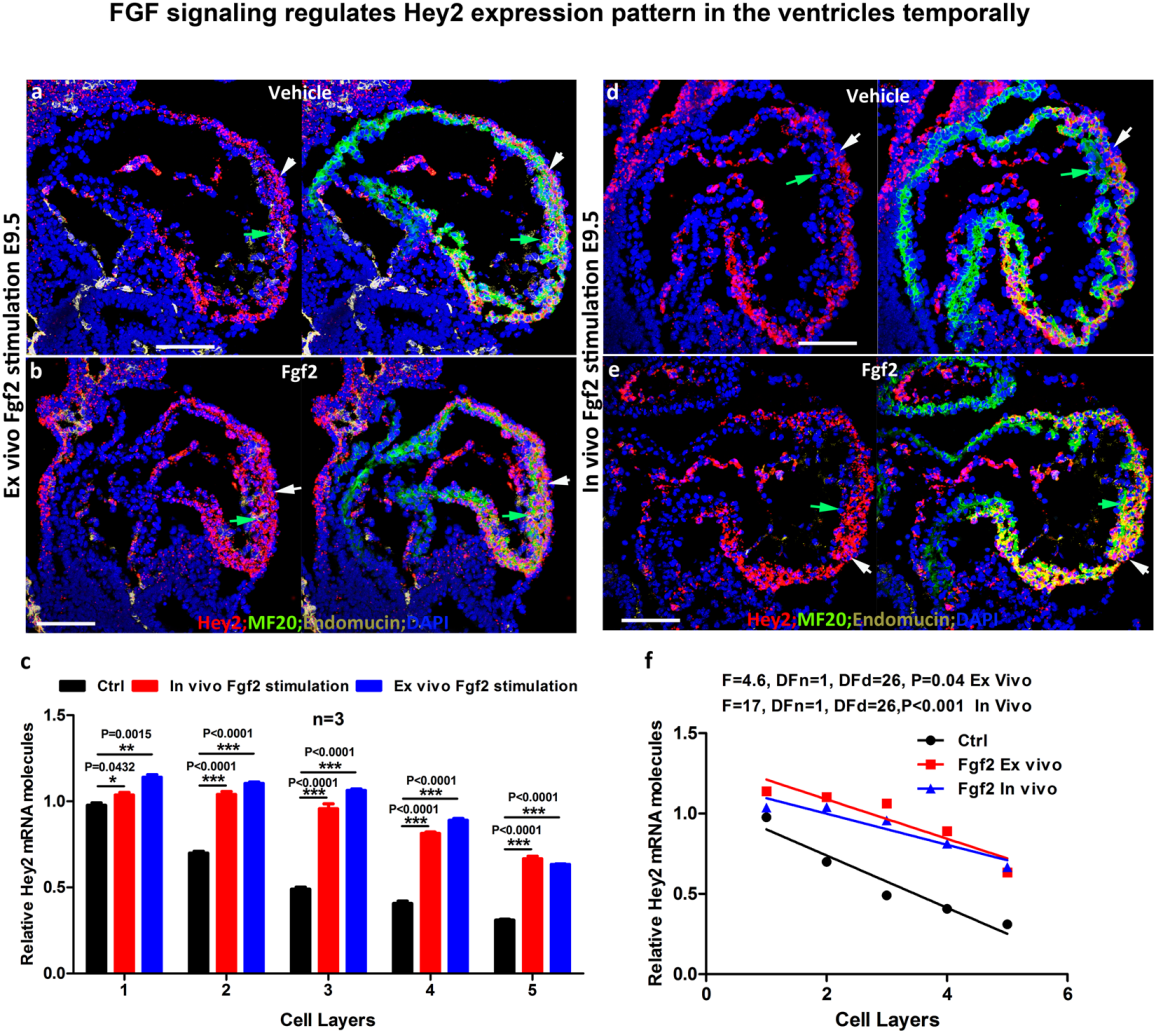
FGF signaling regulates *Hey2* expression pattern in the ventricles temporally. (a and b) FGF2 stimulation increases *Hey2* expression in the inner compact zone as indicated by green arrows in the *ex vivo* cultured heart. **(c)** Quantification and comparison of *Hey2* expression in cardiomyocytes at different layer of *in vivo* and *ex vivo* cultured E9.5 hearts with and without FGF2 stimulation. (d and e) FGF2 stimulation *in vivo* increases *Hey2* expression in the inner compact zone as indicated by green arrows compared to the vehicle treated hearts. (**f**) *Hey2* expression patterns of FGF2 stimulated hearts are significantly different from control hearts based on the linear regression comparison assay. Scale bars in (**a**–**e**) are 100 μm. Representative pictures from at least three embryos of each treatment were shown.

The studies above showed that the signaling pathways such as Notch and Nrg1 signaling that regulate trabeculation and/or compaction control the expression levels but not the expression pattern of *Hey2*. Indeed, the signaling pathway that regulates *Hey2* expression pattern in the myocardium is unknown. In other developmental processes, such as somitogenesis, *Notch* regulates *Hey2*^19^, while in the organ of Corti, FGF signaling regulates *Hey2* expression via a Notch-independent mechanism to maintain pillar cell fate^29^. During cardiac development, FGF2, FGF9, and FGFR1 are the major fibroblast growth factors and receptor that are expressed in mouse and chick ventricles^30–32^. *Fgfr1* and *Fgfr2* cardiac-specific double knockout hearts show increased expression of sarcomeric actin and premature differentiation, indicating that FGF signaling functions to maintain the primitive status of cardiomyoblast^32^. Whether FGF signaling contributes to the relative immaturity of cardiomyocytes in the compact zone and more mature cardiomyocytes in the trabecular zone is not clear. Our RNAscope and immunofluorescence staining show that *Fgfr1* is expressed in the myocardium and endocardium (Suppl. Figure 4a). Considering that multiple FGF receptors and FGF ligands are expressed in the heart, as well as the redundant functions of these ligands and receptors, we examined the effects of FGF signaling on *Hey2* expression pattern by stimulating the hearts with FGF2 *in vivo* and *ex vivo*, instead of using a loss-of-function approach^33^. *Ex vivo* cultured hearts treated with FGF2 ligand for 2 hours showed an increased expression of *Hey2* compared to the control based on the number of *Hey2* mRNAs per cell (Fig. 6a,b). The FGF2 treated hearts also showed enrichment of *Hey2* in both outer compact and inner/trabecular zones compared to the control (Fig. 6b,c). We then further examined if FGF2 stimulation *in vivo* would increase the *Hey2* expression level and change its expression pattern by injecting FGF2 at a concentration of 20ng/gram body weight to the pregnant females when the embryos were at E8.5. We found that FGF2 stimulation increased the expression of pAkt and pErk compared to the vehicle treated control (Suppl. Figure 4b–e), indicating an effective FGF2 stimulation. We then quantified the *Hey2* expression level and examined the enrichment of *Hey2* in the compact zone in both *in vivo* and *ex vivo* models. We found that FGF2 stimulation increased the expression level of *Hey2* and also changed the enrichment of *Hey2* in the compact zone (Fig. 6c–f). The FGF2 induced expression pattern change was not maintained at later stages, as the *Hey2* expression pattern in hearts treated by injecting FGF2 into the pregnant females for three consecutive days was not different from the expression pattern in vehicle treated controls. However, we did find that the FGF2 stimulation promotes trabeculation (Suppl. Figure 4f).

### FGF2 regulates Hey2 expression level in a Notch1 dependent manner

**Figure 7:**
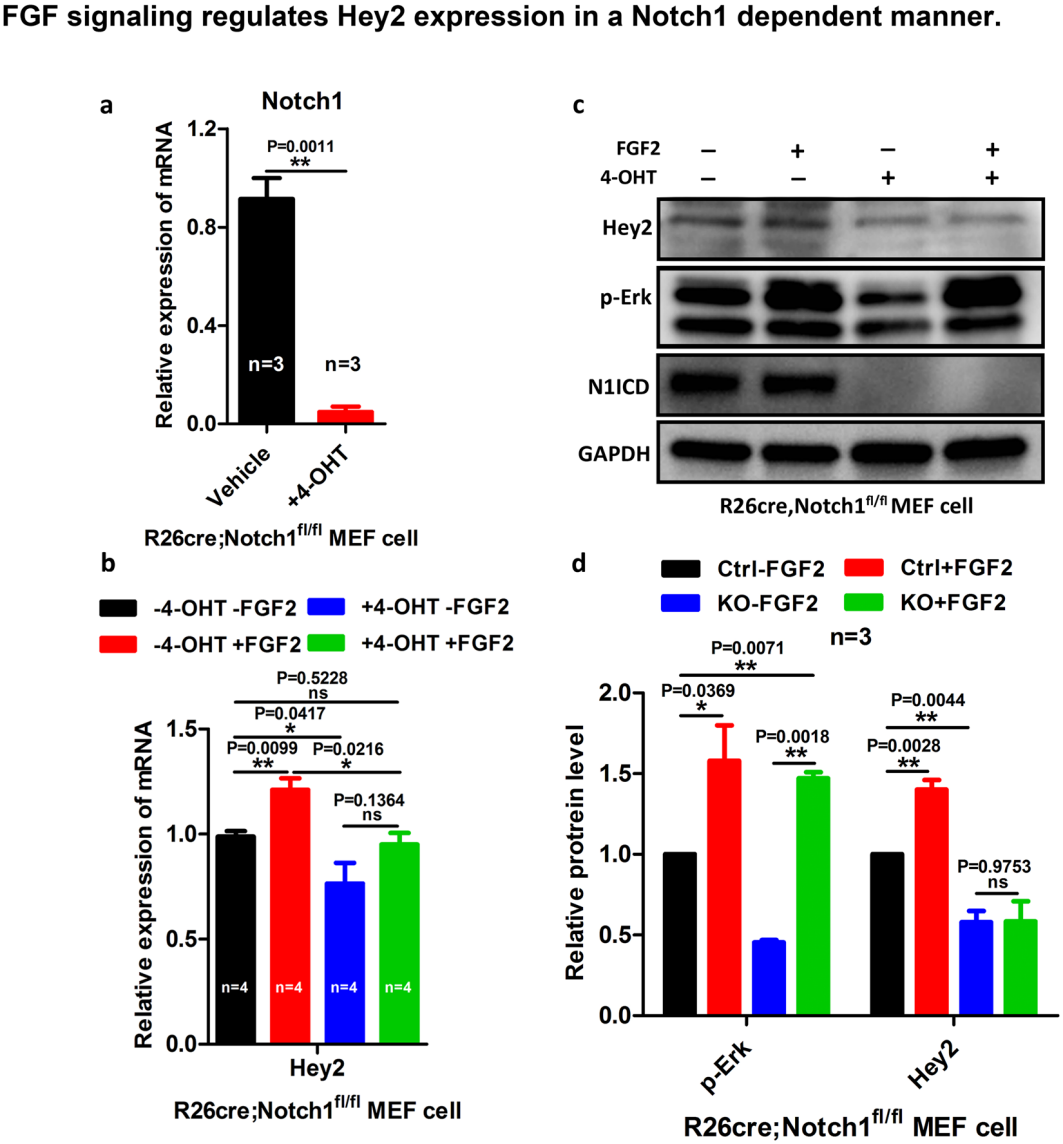
FGF signaling regulates *Hey2* expression in a Notch1 dependent manner. (**a)** *Notch1* is deleted efficiently in *ROSA26*^*CreERT2*^*; Notch1*^*fl/fl*^ MEFs via 4-OHT treatment. **(b)** Q-PCR analysis shows that *Hey2* mRNA level is increased with FGF2 stimulation in control MEF cell and decreased in *Notch1* null MEF cell, while FGF2 stimulation doesn’t cause significant change in *Notch1* null MEFs compared with control based on student’s t-test. (c and d) *Notch1* is deleted efficiently in *ROSA26*^*CreERT2*^*; Notch1*^*fl/fl*^ MEFs by 4-OHT treatment. p-Erk protein level decreased in *Notch1* null MEFs. FGF2 stimulation increases p-Erk protein level in control MEFs but not in *Notch1* null MEFs. FGF2 stimulation promoted Hey2 protein level in control MEFs but not *Notch1* null MEFs. An unpaired, two-tailed student’s t-test was used for statistical comparison. The Representative pictures from three repeats were shown and the original pictures of all the blots are shown in Suppl. Figure 5.

We then used mouse embryonic fibroblasts (MEF) from *ROSA26*^*CreERT2*^*; Notch1*^*fl/fl*^ embryos to further determine whether FGF2 and Notch1 regulate *Hey2* expression level (Fig. 7a–d, Suppl. Figure 5). *ROSA26*^*CreERT2*^*; Notch1*^*fl/fl*^ MEF cells were either treated with vehicle or 4-Hydroxytamoxifen (4-OHT) to delete *Notch1*. qRT-PCR and western blot analyses indicated that the deletion of *Notch1* is efficient (Fig. 7a,c,d). We stimulated the control and *Notch1* null MEFs with FGF2, and the stimulation resulted in the higher level of pErk as expected^33^ (Fig. 7c). We then determined whether FGF2 and *Notch1* regulate the expression level of *Hey2*. *Notch1* deletion decreased the expression of *Hey2* based on Q-PCR and Western blot analyses (Fig. 7b–d). FGF2 stimulation promotes *Hey2* expression in control MEF, but not in the *Notch1* null MEF (Fig. 7b–d). These data suggest that *Notch1* regulates *Hey2* expression and FGF2 regulates *Hey2* in a *Notch1* dependent manner.

## Discussion

### *Hey2* expression in endocardial cells of AVC, OFT and trabeculae might explain the valvular defects of Hey2 mutants

*Hey2*, *Hey1*, and *HeyL* comprise a subfamily of mammalian hairy/enhancer of split-related basic helix-loop-helix genes with a putative *Drosophila* homologue, *dHey*^34^, and a zebrafish homologue, Gridlock^11^. *Hey2* displays a spatiotemporal expression pattern and is expressed in the somite, heart, craniofacial region, and nervous system at early embryonic stages^34^. Detailed examination of *Hey2* expression in mouse heart shows that *Hey2* is expressed in the ventricular septum and compact zone but not in the atrium at all. In this study, we used RNAscope and *Hey2*^*CreERT2*^ knockin mouse line to further characterize *Hey2* expression level and pattern and found that *Hey2* is also expressed in the endocardial cells of the AVC, OFT and at the base of trabeculae (Figs 1 and 2). With the RNAscope and immunofluorescent staining, we can infer that the expression of *Hey2* detected in the cardiac OFT and aortic arch arteries^1,34^ was actually in the endothelial cells (Figs 1 and 2). The expression pattern of *Hey2* via *Hey2*^*CreERT2*^ knockin mouse line is consistent with the results from RNAscope. The expression of *Hey2* in the endocardial cells of the AVC and OFT and mesenchymal cells derived from those endocardial cells might explain the atrioventricular valve defects, pulmonary stenosis, tetralogy of Fallot, and tricuspid atresia found in the *Hey2* knockout mice^7,8^. The expression of *Hey2* in the ventricles but not in atria might explain why the *Hey2* knockouts display ectopic atrial gene expression in the myocardium^12,13^. *Hey2* null cardiomyocytes display malformed mitochondria, abnormal accumulation of glycogen particles, and disorganized myofibrils based on transmission electron microscope analysis^8^, suggesting that *Hey2* might regulate cardiomyocyte differentiation and maturation, and is therefore critical for normal cardiac function. In summary, using RNAscope and *Hey2*^*CreERT2*^ knockin line, we have determined that the *Hey2* expression pattern is broader than previously reported and the expression of *Hey2* in the endocardial cells of the AVC and OFT might explain the variety of defects found in *Hey2* knockout mice and human patients that bear *Hey2* duplication or mutations.

### Notch regulates *Hey2* expression in endocardial cells

Despite *Hey2*’s high homology to other Hey family members^18^, the notion that Notch signaling regulates *Hey2* expression is controversial. During mouse somitogenesis, *Hey2* expression was altered in both the *Dll1* and the *Notch1* knockout mice^19^. NICD overexpression up-regulates *Hey2* in an Rbpjk dependent manner in smooth muscle cells^35^, indicating that Notch signaling regulates *Hey2* expression. However, in the heart, NICD overexpression activated *Hey1* but not *Hey2* expression in the NICD transgenic line^20^, the *Notch2* knockout displayed a normal expression pattern of *Hey1* and *Hey2*^21^, and *Rbpjk* global knockout embryos did not alter the expression of *Hey2* in the heart based on Q-PCR^22^. In addition, in the *Numb* and *Numblike* double knockout heart, in which Notch signaling was upregulated based on a Notch transgenic reporter and N1ICD protein levels^36–38^, there was no obvious *Hey2* up-regulation based on mRNA deep sequencing and Q-PCR. These reports suggest that Notch does not regulate *Hey2* in the heart. However, it is possible that Notch regulates *Hey2* expression in some cell types that cannot be detected by total RNA in the heart or by traditional ISH. In this study, equipped with RNAscope coupled with immunofluorescence staining, we clearly demonstrated that *Hey2* is expressed in endocardial cells as we discussed above. We used *Nfatc1*^*Cre*^ or *Tie2Cre* to delete *Notch1* or *Rbpjk* in the endocardial cells and found that *Hey2* expression was reduced in endocardial cells that burrowed into the myocardium and closely contacted the cardiomyocytes. The expression of *Hey2* in the endocardial cells of the AVC but not of the OFT was slightly reduced in the knockout compared to the control, indicating that Notch signaling at least partially regulates *Hey2* in the endocardial cells of the AVC and those that contact the myocardium. This is consistent with the report that Notch1 is required for proper development of the semilunar valves and cardiac outflow tract^39,40^. Furthermore, the *Hey2* expression in the endocardial cells attached to the myocardium or at the base of the trabecula is higher than that of other endocardial cells, which is consistent with the previous report that NICD is highly expressed in the endocardial cells at the base of the trabecula^24^. The *Hey2* expression in these endocardial cells and its potential to be a target of Notch signaling might explain the trabeculation defect of the *Hey1* and *Hey2* double knockout^41^. Also, the higher expression of *Hey2* and NICD in the endocardial cells attached to cardiomyocytes^24^ indicates a potential Notch signaling pathway from myocardium to endocardium; however, further work needs be done to identify the Notch ligand that regulates *Hey2* expression in the endocardial cells.

### *Hey2* is essential for cardiomyocyte maturation in the left ventricle

The essential functions of *Hey2* in the heart are revealed by the *Hey2* loss of function studies. The *Hey2* null hearts display a dilated left ventricular chamber with markedly diminished fractional shortening of the left ventricle^8^. *Hey2* null cardiomyocytes displayed abnormal mitochondria, abnormal accumulation of glycogen particles, and disorganized myofibrils based on transmission electron microscope analysis^8^. *Hey2* knockouts show ectopic expression of *Tbx5*, *ANF*, and *Cx40* in the compact zone^12,13^. Furthermore, the expression levels of β-MHC and ANF genes in the *Hey2* knockout are increased^8,12^, suggesting that *Hey2* is required to repress atrial gene expression and to maintain ventricular identity. Other studies show that *Hey2* null cardiomyocytes display altered action potentials and mild conduction system expansion^42^, and *Hey2* null cardiomyocytes display defective myocardial calcium release and diminished fractional shortening in the setting of normal calcium stores and calcium sensitivity^43^. It was also reported that the *Hey1* and *Hey2* double knockout displayed a trabeculation defect^41^, and that *Hey1* and *Hey2* might function redundantly to prevent trabecular cardiomyocyte apoptosis^41^. These studies indicate that *Hey2* might be involved in cardiomyocyte maturation during heart development. However, the functions of *Hey2* enrichment in the compact zone but not in the trabecular zone remain unknown.

### Pathways that regulate asymmetric distribution of Hey2 to compact zone and trabecular zones are unknown

Trabecular and compact cardiomyocytes display different features with trabecular cardiomyocytes exhibiting a lower proliferation rate and being more molecularly mature than cardiomyocytes of the compact zone^44^. Trabecular and compact zones can be distinguished by the differential expression of many genes. For example, *p57*, *Irx3*, *BMP10*, Sphingosine 1-phosphate receptor-1 and *Cx40* are highly expressed in the trabecular zone, while *Tbx20*, *Hey2*, and *N-Myc* are highly expressed in the compact zone^14,15,45–48^. Our previous work has shown that asymmetric expression of *Hey2* and *Bmp10* occurs after but not before cytokinesis in perpendicularly oriented cell division, and that their asymmetric distributions are due to the differences in geometric location between the two daughter cells^15^. The daughter cell closer to cardiac jelly displays less *Hey2* and more *BMP10* compared to the other daughter cell that is relatively closer to the surface of the heart, indicating that potential instructive cues for trabecular regional specification might lie in the cardiac jelly and endocardium^15^. Indeed, the signaling pathway that regulates the asymmetric distribution of *Hey2* or other genes between the two zones is unknown. We have showed that Numb Family Proteins regulate Irx3 expression pattern in the heart, but whether Numb also regulates Hey2 is not clear^36,37^. Previous work has shown that at later stages, direct Notch ligand/receptor interactions might be involved in the regulation of trabecular cardiomyocyte differentiation, as overexpression of glycosyltransferase manic fringe in the endocardium can cause abnormal expression patterns of *Hey2* at later gestation stage^49^. However, the deletion of *Notch1* or *Rbpjk* at early stage in endocardial cells or myocardium during trabecular formation did not affect *Hey2* asymmetric distribution. Surprisingly, the signaling pathways that regulate trabecular initiation, including NRG1/ErbB2,4^26-28^, Notch signaling^24^, Hand2^25^ and YAP^50,51^, regulate the expression level of *Hey2*, but not the expression pattern of *Hey2*. This indicates that trabecular initiation and trabecular specification are two different processes and are regulated by different pathways. Instead, we found that FGF2 stimulation regulates *Hey2* asymmetric distribution in myocardium, and results in over-trabeculation, but the mechanisms of how FGF2 regulates *Hey2* and trabeculation will need further studies.

## Methods

### Experimental animals

Mouse strains *Notch1*^52^ was purchased from Jackson Lab. *Nfatc1*^*Cre/*+^, which is specifically expressed in the endocardial cells^23^, was used to delete *Notch1*^52^ and *Rbpjk*^53^ in endocardial cells. *Tie2-Cre*^54^, which is specifically expressed in endothelial cells, was used to delete *Notch1* and *Rbpjk* in endothelial cells. *Nkx2*.*5*^*Cre/+*^ mice^55^, a gift from Dr. Robert Schwartz, were used to delete *Rbpjk*, *Notch1* and *ErbB2*^56^. Male heterozygous with *Nkx2*.*5*^*Cre/+*^ were setup to cross homozygous females to generate knockouts, e.g., *Nkx2*.*5*^*Cre/+*^*; Notch1*^*fl/+*^ males were mated to *Notch1*^*fl/fl*^ females to generate *Nkx2*.*5*^*Cre/+*^; *Notch1*^*fl/fl*^. Their sibling embryos were designated as controls. Dr. Bin Zhou at Shanghai Institutes, Chinese Academy of Sciences, Shanghai, China, provided the *Hey2*^*CreERT2*^ mouse^17^. To isolate embryos, the pregnant females at the specified age will be euthanized by CO2 inhalation and cervical dislocation. CO2 with a filling rate of about 20% of the chamber volume per minute was added to the existing air in the chamber to achieve a balanced gas mixture to fulfill the objective of rapid unconsciousness with minimal distress to the animals. All animal experiments were approved by the Institutional Animal Care and Use Committee (IACUC) at Albany Medical College and performed according to the NIH Guide for the Care and Use of Laboratory Animals.

### RNAscope: *In Situ* hybridization and immunofluorescence staining

*In Situ* hybridization (ISH) and immunofluorescence staining (IFS) were performed according to the protocol of the RNAscope^®^ Chromogenic (RED) Assay (Cat. No. 310036), which can detect single mRNA molecules^15^. Briefly, 24 hours after fixation, the embryos were frozen embedded in OCT compound. Sections of the frozen embedded samples were processed following the step-by-step protocol of the kit. The expressional level of mRNA in each cell was based on the number of mRNA molecules or signal intensity detected using the confocal scanned pictures, and three sections for each genotype were quantified. The number of mRNA molecules in cells at comparable locations was also quantified, and only the control and knockout samples that came from the same litter and underwent the same experiments were quantified and compared.

### Quantification of *Hey2* mRNA molecules in cardiomyocytes at different layers

Cardiomyocytes at different layers starting from the outer compact layer, to the inner compact zone and trabecular zone were selected to quantify the number of *Hey2* molecules. For each sample, we quantified 3 sections, and for each section, 12 cells at 3 different regions per layer were quantified. The mean value of the 36 cells in each layer was presented. Only control and mutants that came from the same litter and underwent the same experiments were quantified and compared. Each experiment was repeated three times.

### MEFs isolation and western blot analysis

Mouse embryonic fibroblasts (MEF) were isolated from E13.5-E14.5 embryos with the genotype of *Notch1*^*fl/fl*^*; ROSA26*^*CreERT2*^. Cultured MEFs were infected treated with 4-OHT as indicated in different experiments for 36 hours, and then starved with serum free medium overnight. The cells were then stimulated with FGF2 ligand for 15 minutes. After the stimulation, the cell lysate was harvested. Lysates from MEFs of different genotypes and treatment were processed for western blot. Antibodies used for western blots were listed above. The experiments were repeated three times and quantification data was shown in the figure.

### Paraffin and frozen section immunohistochemistry (IHC)

Immunohistochemistry (IHC) and Immunofluorescence (IF) were performed as previously published^57–59^. The following primary antibodies were used: BrdU (1:50, Becton Dickinson, 347583), PECAM (1:50, BD Pharmingen), MF20 (1:100, Developmental Studies Hybridoma Bank (DSHB)), NICD (1:50, Cell Signaling, 4147 S), ErbB2 (1:100, Cell Signaling, 2165 S), ESR (Abcam, pre-diluted), Endomucin (1:50, SCBT), p-Akt (1:50, Cell Signaling, 9271 S) and p-Erk (1:100, Cell Signaling, 4376 S).

### *Ex vivo* culture system

The *ex vivo* culture protocol was developed by Dr. C.P. Cheng and was utilized as reported^36^. Briefly, embryos lacking the head and tail were cultured in medium containing FGF2 at 2ng/gram body weight or vehicle for 2 hours. Then, the embryos were fixed and used for RNAscope.

### Imaging

The following systems were used: for confocal imaging, Zeiss LSM880-META NLO confocal microscope equipped with Airyscan; for color imaging, Zeiss Observer Z1 with a Hamamatsu ORCA-ER camera; for fluorescence imaging, Zeiss Observer Z1 with an AxioCam MRm camera. All histology analysis and immune-fluorescent staining analysis were quantitated in a blinded way by at least two investigators.

### Statistics

Data is shown as mean ± standard deviation. An unpaired, two-tailed student’s t-test or linear regression comparison as specified were used for statistical comparison. A P-value of 0.05 or less was considered statistically significant.

## Electronic supplementary material

Supplemental Dataset

## References

[1] Nakagawa O, Nakagawa M, Richardson JA, Olson EN, Srivastava D (1999). HRT1, HRT2, and HRT3: a new subclass of bHLH transcription factors marking specific cardiac, somitic, and pharyngeal arch segments. Dev Biol.

[2] Steidl C (2000). Characterization of the human and mouse HEY1, HEY2, and HEYL genes: cloning, mapping, and mutation screening of a new bHLH gene family. Genomics.

[3] Kageyama R, Nakanishi S (1997). Helix-loop-helix factors in growth and differentiation of the vertebrate nervous system. Curr Opin Genet Dev.

[4] Reamon-Buettner SM, Borlak J (2006). HEY2 mutations in malformed hearts. Human mutation.

[5] Thorsson T (2015). Chromosomal Imbalances in Patients with Congenital Cardiac Defects: A Meta-analysis Reveals Novel Potential Critical Regions Involved in Heart Development. . Congenital heart disease.

[6] Jordan VK, Rosenfeld JA, Lalani SR, Scott DA (2015). Duplication of HEY2 in cardiac and neurologic development. Am J Med Genet A.

[7] Donovan J, Kordylewska A, Jan YN, Utset MF (2002). Tetralogy of fallot and other congenital heart defects in Hey2 mutant mice. Curr Biol.

[8] Kokubo H (2004). Targeted disruption of hesr2 results in atrioventricular valve anomalies that lead to heart dysfunction. Circ Res.

[9] Sakata Y (2006). The spectrum of cardiovascular anomalies in CHF1/Hey2 deficient mice reveals roles in endocardial cushion, myocardial and vascular maturation. J Mol Cell Cardiol.

[10] Bezzina CR (2013). Common variants at SCN5A-SCN10A and HEY2 are associated with Brugada syndrome, a rare disease with high risk of sudden cardiac death. Nat Genet.

[11] Zhong TP, Rosenberg M, Mohideen MA, Weinstein B, Fishman M (2000). C. gridlock, an HLH gene required for assembly of the aorta in zebrafish. Science.

[12] Xin M (2007). Essential roles of the bHLH transcription factor Hrt2 in repression of atrial gene expression and maintenance of postnatal cardiac function. Proc Natl Acad Sci USA.

[13] Koibuchi N, Chin MT (2007). CHF1/Hey2 plays a pivotal role in left ventricular maturation through suppression of ectopic atrial gene expression. Circ Res.

[14] Sedmera D, Pexieder T, Vuillemin M, Thompson RP, Anderson RH (2000). Developmental patterning of the myocardium. Anat Rec.

[15] Li J (2016). Single-Cell Lineage Tracing Reveals that Oriented Cell Division Contributes to Trabecular Morphogenesis and Regional Specification. Cell reports.

[16] Kokubo H, Miyagawa-Tomita S, Johnson RL (2005). Hesr, a mediator of the Notch signaling, functions in heart and vessel development. Trends Cardiovasc Med.

[17] Tian X (2017). Identification of a hybrid myocardial zone in the mammalian heart after birth. Nature communications.

[18] Iso T, Kedes L, Hamamori Y (2003). HES and HERP families: multiple effectors of the Notch signaling pathway. Journal of cellular physiology.

[19] Leimeister C (2000). Oscillating expression of c-Hey2 in the presomitic mesoderm suggests that the segmentation clock may use combinatorial signaling through multiple interacting bHLH factors. Dev Biol.

[20] Watanabe Y (2006). Activation of Notch1 signaling in cardiogenic mesoderm induces abnormal heart morphogenesis in mouse. Development.

[21] Kokubo H, Tomita-Miyagawa S, Hamada Y, Saga Y (2007). Hesr1 and Hesr2 regulate atrioventricular boundary formation in the developing heart through the repression of Tbx2. Development.

[22] Timmerman LA (2004). Notch promotes epithelial-mesenchymal transition during cardiac development and oncogenic transformation. Genes Dev.

[23] Wu B (2012). Endocardial cells form the coronary arteries by angiogenesis through myocardial-endocardial VEGF signaling. Cell.

[24] Grego-Bessa J (2007). Notch signaling is essential for ventricular chamber development. Dev Cell.

[25] VanDusen NJ (2014). Hand2 is an essential regulator for two Notch-dependent functions within the embryonic endocardium. Cell reports.

[26] Gassmann M (1995). Aberrant neural and cardiac development in mice lacking the ErbB4 neuregulin receptor. Nature.

[27] Lee KF (1995). Requirement for neuregulin receptor erbB2 in neural and cardiac development. Nature.

[28] Meyer D, Birchmeier C (1995). Multiple essential functions of neuregulin in development. Nature.

[29] Doetzlhofer A (2009). Hey2 regulation by FGF provides a Notch-independent mechanism for maintaining pillar cell fate in the organ of Corti. Dev Cell.

[30] Pennisi DJ, Ballard VL, Mikawa T (2003). Epicardium is required for the full rate of myocyte proliferation and levels of expression of myocyte mitogenic factors FGF2 and its receptor, FGFR-1, but not for transmural myocardial patterning in the embryonic chick heart. Dev Dyn.

[31] Colvin, J. S., Feldman, B., Nadeau, J. H., Goldfarb, M. & Ornitz, D. M. Genomic organization and embryonic expression of the mouse fibroblast growth factor 9 gene. Dev Dyn **216** 72–88, https://doi.org/10.1002/(SICI)1097-0177(199909)216:1<72::AID-DVDY9>3.0.CO;2-9 (1999). 10.1002/(SICI)1097-0177(199909)216:1<72::AID-DVDY9>3.0.CO;2-9 10474167

[32] Lavine KJ (2005). Endocardial and epicardial derived FGF signals regulate myocardial proliferation and differentiation *in vivo*. Dev Cell.

[33] Li J (2017). CDC42 is required for epicardial and pro-epicardial development by mediating FGF receptor trafficking to the plasma membrane. Development.

[34] Leimeister C, Externbrink A, Klamt B, Gessler M (1999). Hey genes: a novel subfamily of hairy- and Enhancer of split related genes specifically expressed during mouse embryogenesis. Mech Dev.

[35] Iso T, Chung G, Hamamori Y, Kedes L (2002). HERP1 is a cell type-specific primary target of Notch. J Biol Chem.

[36] Zhao C (2014). Numb family proteins are essential for cardiac morphogenesis and progenitor differentiation. Development.

[37] Wu M, Li J (2015). Numb family proteins: novel players in cardiac morphogenesis and cardiac progenitor cell differentiation. Biomolecular concepts.

[38] Yang, J. *et al*. Inhibition of Notch2 by Numb/Numblike controls myocardial compaction in the heart. Cardiovasc Res, 10.1093/cvr/cvs250 (2012). 10.1093/cvr/cvs250 22865640

[39] Koenig, S. N. *et al*. Endothelial Notch1 Is Required for Proper Development of the Semilunar Valves and Cardiac Outflow Tract. Journal of the American Heart Association **5**, 10.1161/JAHA.115.003075 (2016). 10.1161/JAHA.115.003075 PMC4843530 27107132

[40] Garg V (2005). Mutations in NOTCH1 cause aortic valve disease. Nature.

[41] Kokubo H, Miyagawa-Tomita S, Nakazawa M, Saga Y, Johnson RL (2005). Mouse hesr1 and hesr2 genes are redundantly required to mediate Notch signaling in the developing cardiovascular system. Dev Biol.

[42] Hartman ME (2014). Myocardial deletion of transcription factor CHF1/Hey2 results in altered myocyte action potential and mild conduction system expansion but does not alter conduction system function or promote spontaneous arrhythmias. FASEB J.

[43] Liu Y (2012). Transcription factor CHF1/Hey2 regulates EC coupling and heart failure in mice through regulation of FKBP12.6. Am J Physiol Heart Circ Physiol.

[44] Sedmera D (2003). Spatiotemporal pattern of commitment to slowed proliferation in the embryonic mouse heart indicates progressive differentiation of the cardiac conduction system. Anat Rec A Discov Mol Cell Evol Biol.

[45] Zhang W, Chen H, Qu X, Chang CP, Shou W (2013). Molecular mechanism of ventricular trabeculation/compaction and the pathogenesis of the left ventricular noncompaction cardiomyopathy (LVNC). Am J Med Genet C Semin Med Genet.

[46] Kochilas LK, Li J, Jin F, Buck CA, Epstein JA (1999). p57Kip2 expression is enhanced during mid-cardiac murine development and is restricted to trabecular myocardium. Pediatr Res.

[47] Chen H (2004). BMP10 is essential for maintaining cardiac growth during murine cardiogenesis. Development.

[48] Clay H (2016). Sphingosine 1-phosphate receptor-1 in cardiomyocytes is required for normal cardiac development. Dev Biol.

[49] D’Amato G (2016). Sequential Notch activation regulates ventricular chamber development. Nat Cell Biol.

[50] Xin M (2011). Regulation of insulin-like growth factor signaling by Yap governs cardiomyocyte proliferation and embryonic heart size. Science signaling.

[51] von Gise A (2012). YAP1, the nuclear target of Hippo signaling, stimulates heart growth through cardiomyocyte proliferation but not hypertrophy. Proc Natl Acad Sci USA.

[52] Yang X (2004). Notch activation induces apoptosis in neural progenitor cells through a p53-dependent pathway. Dev Biol.

[53] Han H (2002). Inducible gene knockout of transcription factor recombination signal binding protein-J reveals its essential role in T versus B lineage decision. Int Immunol.

[54] Kisanuki YY (2001). Tie2-Cre transgenic mice: a new model for endothelial cell-lineage analysis *in vivo*. Dev Biol.

[55] Moses KA, DeMayo F, Braun RM, Reecy JL, Schwartz RJ (2001). Embryonic expression of an Nkx2-5/Cre gene using ROSA26 reporter mice. Genesis.

[56] Crone SA (2002). ErbB2 is essential in the prevention of dilated cardiomyopathy. Nat Med.

[57] Mellgren AM (2008). Platelet-derived growth factor receptor beta signaling is required for efficient epicardial cell migration and development of two distinct coronary vascular smooth muscle cell populations. Circ Res.

[58] Lechler T, Fuchs E (2005). Asymmetric cell divisions promote stratification and differentiation of mammalian skin. Nature.

[59] Wu M (2010). Epicardial spindle orientation controls cell entry into the myocardium. Dev Cell.

