# Supplementary material for: Notch signaling regulates Hey2 expression in a spatiotemporal dependent manner during cardiac morphogenesis and trabecular specification
Source: Sci Rep. 2018 Feb 8;8:2678. doi: 10.1038/s41598-018-20917-w (PMC5805758; doi:10.1038/s41598-018-20917-w)
Supplement: Supplementary file 1 — Supplemental Dataset [file 41598_2018_20917_MOESM1_ESM.pdf]

Notch signaling regulates *Hey2* expression in a spatiotemporal dependent manner during cardiac morphogenesis and trabecular specification

Lianjie Miao[1, 2], Jingjing Li[1], Jun Li[1], Xueying Tian[3], Yangyang Lu[1], Saiyang Hu[1], David Shieh[1], Ryan Kanai[1], Boyang Zhou[1], Bin Zhou[4], Jiandong Liu[5], Anthony B. Firulli[6,] James F. Martin[7], Harold Singer[1], Bin Zhou[3], Hongbo Xin[2*] & Mingfu Wu[1*]

[1] Department of Molecular and Cellular Physiology, Albany Medical College, Albany, NY
[2] Institute of Translational Medicine, Nanchang University, Nanchang, China.
School of Life Sciences, Nanchang University, Nanchang, China.
[3]The State Key Laboratory of Cell Biology, Shanghai Institute of Biochemistry and Cell Biology, Chinese Academy of Sciences, University of Chinese Academy of Sciences, Shanghai 200031, China.
[4] Department of Genetics, Albert Einstein College of Medicine of Yeshiva University, Bronx, New York 10461, USA.
[5]Department of Pathology and Laboratory Medicine, McAllister Heart Institute, University of North Carolina at Chapel Hill, NC 27599, USA
[6] Riley Heart Research Center, Wells Center for Pediatric Research, Departments of Pediatrics and Medical and Molecular Genetics, Indiana University, Indianapolis, IN 46202, USA
[7]Department of Molecular Physiology and Biophysics, Baylor College of Medicine, Houston, TX.

*Correspondence to Mingfu Wu, Ph.D.
43 New Scotland Ave,
Department of Molecular and Cellular Physiology,
Albany Medical College, Albany NY 12208
E-mail **wum@mail.amc.edu**
Phone: 518-262-5795
Fax: 518-262-8101

*Correspondence to Hongbo Xin, Ph.D.
The Center for Cardiovascular Diseases
Institute of Translational Medicine
Nanchang University
999 Xuefu Road, Honggu District
Nanchang 330031, P.R.China
E-mail: xinhb@ncu.edu.cn
Tel: 86-791-83969015(Office)
Fax: 86-791-83969015

Key words: Trabeculation, *Hey2* Expression Pattern, Notch Signaling, Endocardial Cells, Numb Family Proteins

Supplemental Figure legends

Suppl Fig.1. Rbpjk independent signaling also regulates *Hey2* expression in endocardial cells.

(a&b) *Hey2* expression in control and *Tie2*$^{cre/+}$*; Notch1*$^{fl/fl}$ hearts at E9.5. White arrows in b1 show reduced *Hey2* expression compared to a1. The box regions in a-b are zoomed to separate pictures that are labeled with the same name of the box. The red arrow in b indicates an incomplete Hey2 abolishment in the endocardial cells of *Tie2*$^{cre/+}$*; Notch1*$^{fl/fl}$. Based on Q-PCR via student's t-test, *Hey2* relative expression level in the whole heart of *Tie2*$^{cre/+}$*; Rbpjk*$^{fl/fl}$ is greater than expression in the control possibly due to less trabeculae and more compact cardiomyocytes. Scale bars in a-b are 50 μm. Representative pictures from at least three embryos of each genotype were shown.

Suppl Fig.2. Notch signaling in myocardium regulates the expression level but not the pattern of *Hey2* in the myocardium at E12.5.

(a&b) *Hey2* expression in control and KO (*Nkx2.5*$^{cre/+}$; *RBPJk*$^{fl/fl}$) hearts at E12.5. *Hey2* is enriched in the compact zone in both the control and the KO heart, but the expression level of *Hey2* in the KO is decreased. (c) Based on Q-PCR, *Hey2* relative expression level in the whole heart of *Nkx2.5*$^{cre/+}$*; Rbpjk*$^{fl/fl}$ is lower than in the control. Scale bars in a-b are 100 μm. Representative pictures from at least three embryos of each genotype were shown.

Suppl Fig.3. Hey2 expression was reduced in *Nkx2.5*$^{cre/+}$*; ErbB2*$^{fl/fl}$ hearts at E9.5.

(a) *Hey2* expression in different cell layers was quantified and *Nkx2.5*$^{cre/+}$*; ErbB2*$^{fl/fl}$. KO hearts displayed a lower expression of *Hey2* compared to controls, but the *Hey2* expression pattern was not changed. Representative pictures from at least three embryos of each genotype were shown.

Suppl Fig.4. FGF2 stimulation does not disrupt *Hey2* expression pattern at E12.5.

(a) *Fgfr1* expression via ISH in cardiomyocytes in an E9.5 heart indicated by the white arrows. **(b&c)** FGF2 stimulation increased the level of pAkt compared to the vehicle treated control. **(d&e)** show that FGF2 stimulation increased the expression of pErk compared to the vehicle treated control. **(f)** FGF2 stimulation by consecutively injecting FGF2 to the pregnant females for three days doesn't change the *Hey2* expression pattern at E12.5. Scale bars in a-e are 100 μm, and 50 μm in f. Representative pictures from at least three embryos of each treatment were shown.

Supplemental table 1
Q-PCR primers for mouse samples

Hey2	gtggggagcgagaacaatta	gttgtcggtgaattggacct
Cyclophilin	ggagatggcacaggaggaa	gcccgtagtgcttcagctt
Notch1	ctggaccccatggacatc	aggatgactgcacacattgc

Suppl. Fig.1 Rbpjk independent signaling also regulates Hey2 expression in endcoardial cells.

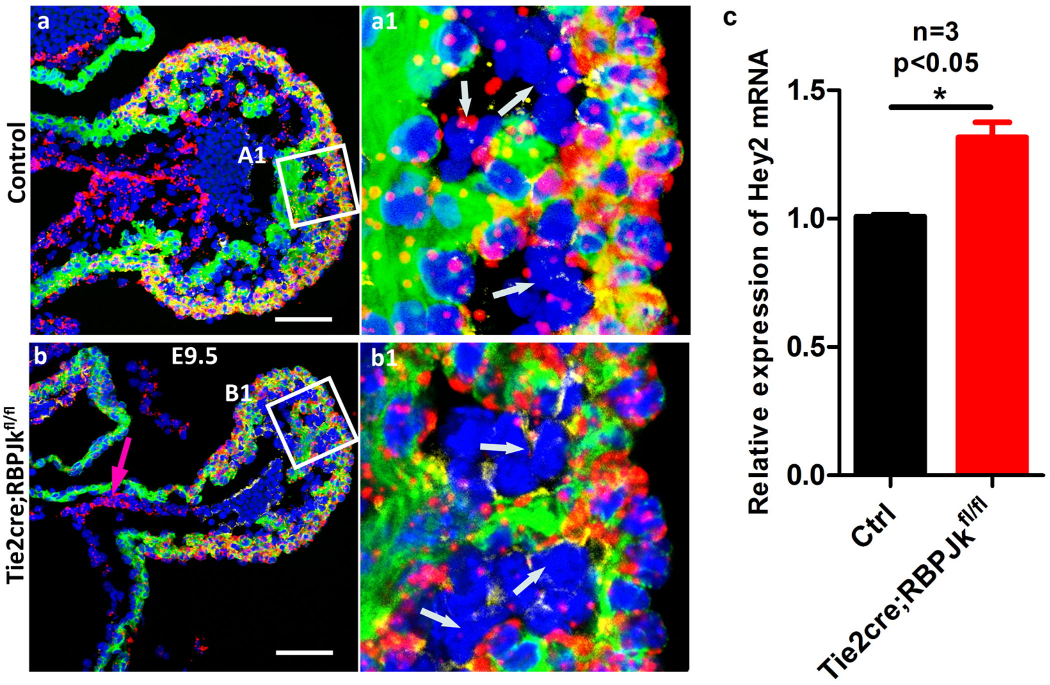

Suppl Fig.2 Notch signaling in myocardium regulates the expression level but not the pattern of Hey2 in the myocardium at E12.5.

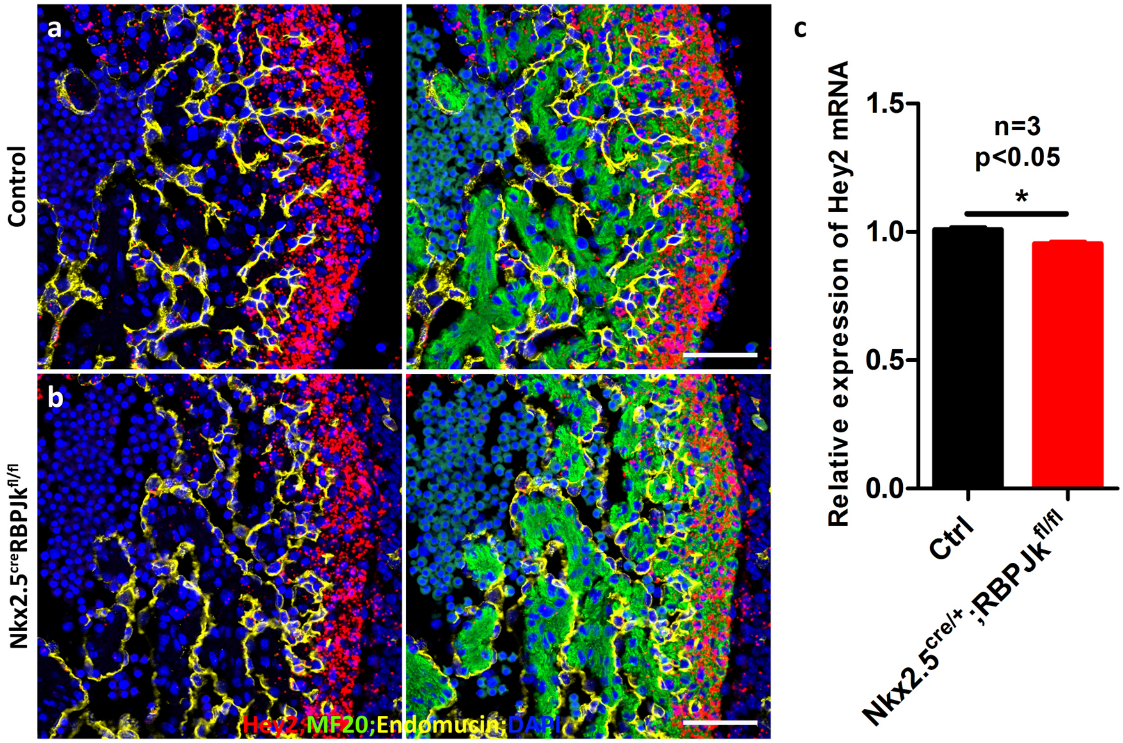

Suppl Fig.3 Hey2 expression was reduced in Nkx2.5$^{cre/+}$; ErbB2$^{fl/fl}$ heart at E9.5.

a

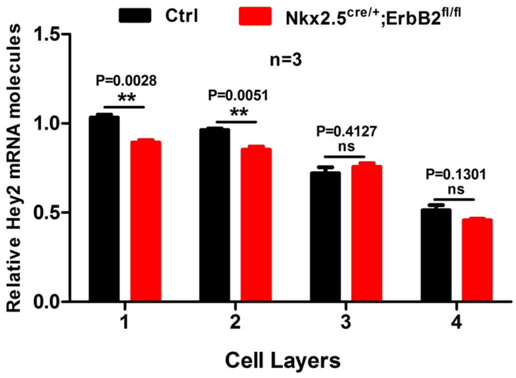

Suppl Fig. 4 FGF2 stimulation does not disrupt Hey2 expresion pattern at E12.5

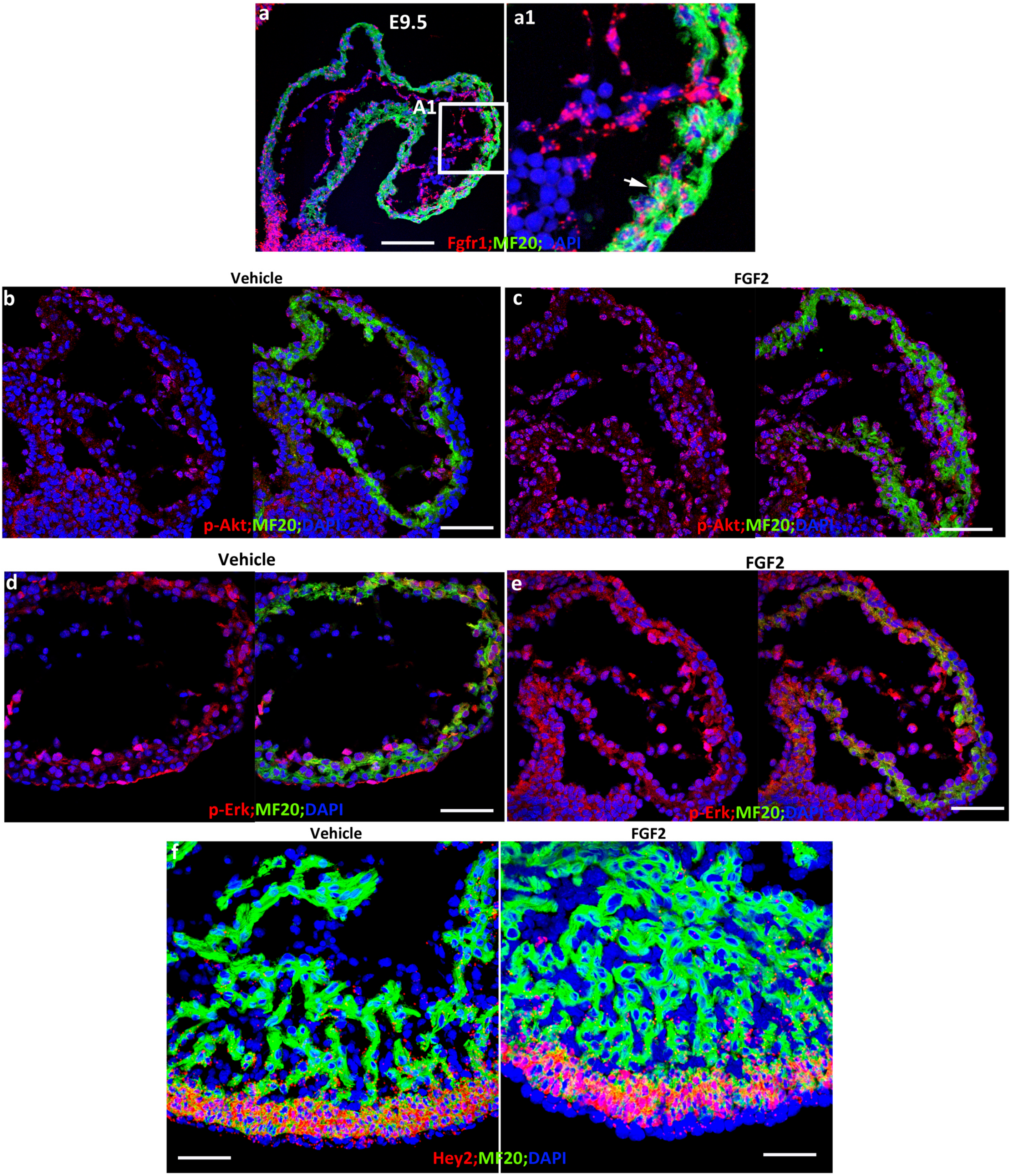

Suppl Fig.5 Raw data for Western blot results in Fig.7.

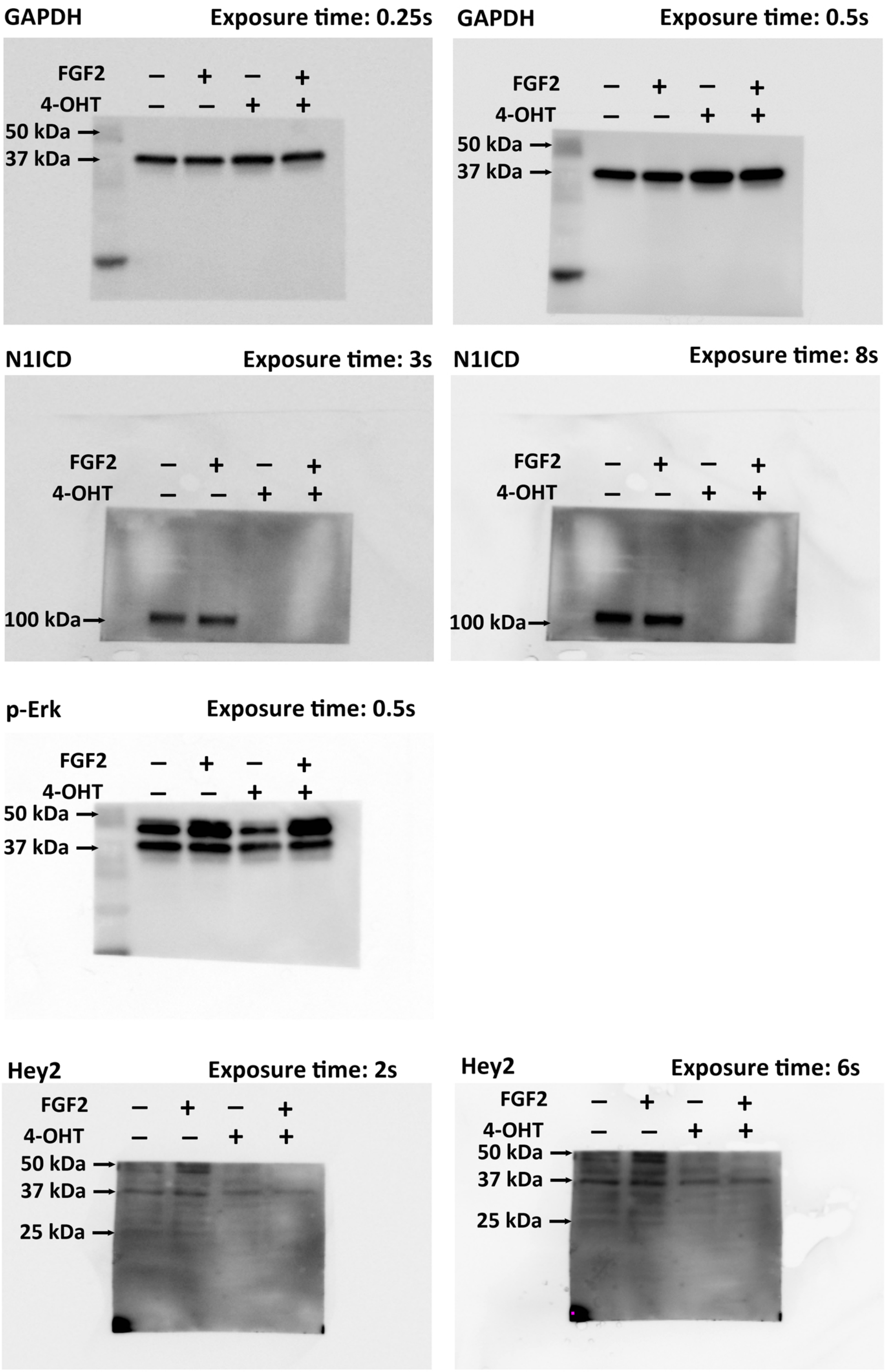

R26cre,Notch1$^{fl/fl}$ MEF cell
